# Draft genome sequence of *Acinetobacter haemolyticus* strain MUWRP1017 isolated from the pus of a female inpatient at Bwera General Hospital in Uganda

**DOI:** 10.1128/mra.00566-24

**Published:** 2024-08-20

**Authors:** Godfrey Wokorach, Bernard Erima, Stephen Alafi, Hope O. Kabatesi, Julius T. Muhindo, Florence Najjuka, James Kiyengo, Hannah Kibuuka, Ambrose K. Musinguzi, Fred Wabwire-Mangen, Denis K. Byarugaba

**Affiliations:** 1 Makerere University Walter Reed Project, Kampala, Uganda; 2 Department of Biology, Faculty of Science, Muni University, Arua, Uganda; 3 Bwera District Hospital, Kasese, Uganda; 4 Makerere University, Kampala, Uganda; 5 Uganda Peoples Defence Forces, Kampala, Uganda; Department of Biological Sciences, Wellesley College, Wellesley, Massachusetts, USA

**Keywords:** *blaOXA-264*, *blaADC-25*, *Acinetobacter*, bacteria, Gram-negative bacteria, Uganda

## Abstract

The bacterium *Acinetobacter haemolyticus*, with a genome size of 3.4 Mb, was isolated from a pus swab of a wound on the left lower limb above the ankle joint of a female patient. This strain carries the antimicrobial resistance genes cephalosporinase *blaADC-25*, oxallinase *blaOXA-264*, *floR*, and *sul2* and other resistance and virulence genes.

## ANNOUNCEMENT

*Acinetobacter is* a group of Gram-negative coccobacillus bacteria with increasing prevalence in hospitals worldwide, causing various infections (1). *Acinetobacter haemolyticus* is an emerging species with serious multidrug resistance agents like *A. baumanii*. It belongs to the haemolytic clade and has been implicated in nosocomial infections, with some carrying carbapenem-resistance determinants, making treatment difficult (2). This study describes the genome of strain MUWRP1017 isolated from a wound on the lower left limb of a female patient from the Democratic Republic of Congo. She received healthcare service at Bwera General Hospital in Kasese district, Uganda.

A swab obtained from pus was immediately inoculated on blood agar, and the resulting single colony was transferred to nutrient agar. The bacterial colony was presumptively identified as Gram-negative coccobacillus by Gram staining and was later grown on Luria broth to obtain cell mass for DNA extraction. All culturing procedures were done at 37°C for 24 hours under aerobic conditions. DNA was extracted using the DNeasy UltraClean microbial kit (Qiagen, Germantown, Maryland, USA) without modification. Extracted DNA was used to create libraries with the Kapa HyperPlus library preparation kit (Roche Diagnostics, Indianapolis, IN, USA). Paired-end sequencing (2 × 151 bp) of the libraries produced 11,677,660 total reads on Illumina NextSeq (Illumina, Inc., San Diego, CA). Raw reads were evaluated using FastQC v0.11.9 (https://www.bioinformatics.babraham.ac.uk/projects/fastqc/) and trimmed with Btrim v0.2.0 (3). Assembly with Newbler v2.7 generated a genome with 523 x coverage (4). The assembled genome was evaluated for completeness using CheckM v1.2.2 (5). The annotation of the draft genome was done using PGAP version 6.7 (6). The taxonomy assignment was done using the Genome Taxonomy Database (GTDB Release 214.1) considering an average nucleotide identity (ANI) index of ≥95% (7). Antimicrobial resistant genes were predicted with ResFinder v4.3.2 (8). The virulence factor database (VFDB) (http://www.mgc.ac.cn/VFs/) was used to predict the presence of virulence genes (9). All software utilized default settings, unless otherwise stated. Ethical approval was obtained from Makerere University School of Public Health Higher Degrees and Research Ethics Committee (HDREC 087) and Walter Reed Army Institute of Research IRB (WRAIR #1711).

CheckM v1.2.2 indicates the assembled genome was 99.99% complete. The assembly generated a genome of 3.4 Mb and 119 contigs with an N50 value of 70 kb. About 39.5% of the genome is made of GC bases. The genome was predicted to have 3,235 genes, of which 3,151 were protein-coding genes. The MUWRP1017 strain was identified as *Acinetobacter haemolyticus,* with the closest match to type strain (MTCC 9819), with an ANI value of 98.13% (Table 1). The MUWRP1017 strain carries *aac(6')-Ig*, *aph (6)-Id*, and *aph(3'')-Ib* aminoglycoside resistant genes. The cephalosporinase *blaADC-25* and *oxallinase blaOXA-264* were the detected beta-lactam-resistant genes. Amphenicol (*floR*) and folate pathway antagonist (*sul2*) resistant genes were also detected. Adherence genes (*pilT*, *pilU*, *pilB*, *mshE*, *tapT*, and *htpB*) and Type VI secretion system tip protein (*vgrG/tssI*) were the virulence genes detected. The finding of *Acinetobacter*-derived cephalosporinase *blaADC-25, the oxallinase blaOXA-264,* and other resistance determinants is an alert for potential resistance threats in future.

**TABLE 1:** Average nucleotide identity (ANI) index of type strains with strain MUWRP1017

Organism name	Type strain	Accession	Taxonomy ID	ANI (%)
*A. haemolyticus*	MTCC 9819	GCA_000430205.1	29430	98.13
*A. haemolyticus*	CIP 64.3	GCA_000369065.1	29430	98.25
*A. haemolyticus*	FDAARGOS_1392	GCA_019355995.1	29430	98.30
*A. haemolyticus*	NCTC10305	GCA_900444835.1	29430	98.30

## Data Availability

This Whole Genome Shotgun project has been deposited at DDBJ/ENA/GenBank under the accession number JBDPJG000000000. The version described in this paper is version JBDPJG010000000. The GenBank assembly accession number is GCA_039839735.1. The raw sequences were deposited in the Sequence Read Archive (SRA) under the accession number SRR29081551.
